# Depressive Symptoms Among Frontline Healthcare Workers in a Public Hospital in Ciudad Juárez, Mexico: A Post-Pandemic Cross-Sectional Study

**DOI:** 10.3390/healthcare14213730

**Published:** 2026-11-03

**Authors:** Miriam Paola Orozco Inoztroza, Perla Yaneli Ruiz-Montes, Ivette Janeth Reveles-Manriquez, Juana Trejo-Franco, Flor Rocío Ramírez-Martínez, Luis Flores-Padilla

**Affiliations:** 1 Department of Nursing, Mexican Social Security Institute, 3970 Anillo Envolvente—PRONAF, Ciudad Juárez 32310, Mexico; mirinoztroza_86@hotmail.com (M.P.O.I.); yarumo27@hotmail.com (P.Y.R.-M.); janetterev@hotmail.com (I.J.R.-M.); 2 Department of Administrative Sciences, Autonomous University of City Juarez, 1210 Plutarco Elias Calles, Ciudad Juárez 32310, Mexico; jtrefra@yahoo.com (J.T.-F.); rocio.ramirez@uacj.mx (F.R.R.-M.)

**Keywords:** depression, COVID-19, healthcare workers, pandemic, nurses

## Abstract

Background: The COVID-19 pandemic was associated with substantial psychological burden among healthcare workers worldwide. Reported prevalences of depressive symptoms range from 21.7% to 64.7%. Nurses and physicians working on the front lines were particularly vulnerable to these psychological changes. This study aimed to describe the severity of depressive symptoms and explore their association with the sociodemographic and occupational characteristics of healthcare workers in a public hospital in Ciudad Juárez, Mexico. Materials and Methods: A cross-sectional observational study was conducted at a public hospital of the Mexican Social Security Institute in Ciudad Juarez Mexico. The sample consisted of 296 healthcare workers providing front-line care to patients with COVID-19. A questionnaire collected sociodemographic and occupational variables, including age, sex, marital status, pre-existing health conditions, job title, academic level, and access to personal protective equipment (PPE). The Beck Depression Inventory II (BDI II) was utilized to evaluate depressive symptoms. Bivariate analysis was performed using the chi-square test (X^2^) with the severity of depressive symptoms. Bivariate analyses were performed using a chi-square test (X^2^), calculating odds ratios (ORs) and confidence intervals (CIs). Binary logistic regression analysis was conducted to explore the independent predictors of depressive symptoms severity. Results: The overall prevalence of depressive symptoms was 38.5%. The presence of depression was significantly associated with pre-existing medical conditions (*p* < 0.00001), anxiety (*p* < 0.00001), female sex (*p* < 0.006), and having self-isolated to protect one’s family (*p* < 0.01). Additionally, being a specialist physician was significantly associated with the risk of severe depressive symptoms (*p* < 0.04). Regarding moderate depressive symptoms, significant associations were found with rarely having access to the cafeteria during the workday (*p* < 0.004), occasionally having access to PPE (*p* < 0.005), and not having personal protective equipment at all (*p* < 0.04). Conclusions: A high prevalence of depressive symptoms was observed among front-line healthcare workers, showing significant statistical associations with female sex, specific medical backgrounds, self-isolation practices, and occupational pathways.

## 1. Introduction

In 2019, the severe acute respiratory syndrome caused by SARS-CoV-2, known as COVID-19, emerged; it spread worldwide and was declared a pandemic in 2020 by the World Health Organization. By March 2021, the SARS-Co-2 virus had spread to more than 219 countries and territories, reaching over 129.7 million confirmed cases and causing more than three million deaths worldwide [1,2]. Since its emergence, this virus has radically changed the world; its impact is evident in the number of infections and deaths [3]. As a result, hospital systems worldwide were overwhelmed, affecting frontline healthcare workers. Considering the vulnerability of our country to the disease, more than 650,000 healthcare workers were on the front lines of the pandemic [4]. Wearing only the personal protective equipment that was strictly necessary, the constant fear of becoming infected or infecting their loved ones, anxiety, frustration caused by being overwhelmed by the number of patients and the progression of the disease, and the fact that healthcare workers are among the most vulnerable groups pushed their mental health to the limit. All of these examples can lead to mental health issues, such as depression, and can affect decision-making and job performance [5]. Depression is characterized by feelings of sadness and a loss of interest in daily activities, as well as symptoms such as loss of energy, changes in eating habits, decreased or increased sleep, difficulty concentrating, indecision, restlessness, feelings of worthlessness, guilt or hopelessness, and suicidal thoughts [6]. Previous studies on respiratory syndromes, such as SARS in 2003, reported cases of depression affecting the mental health of personnel who were on the front lines of this emergency; their concerns included the fear of becoming ill or infecting their loved ones, as well as feelings of uncertainty and stigmatization [7].

The pandemic has been linked to negative psychological effects among healthcare workers; in China, the first country affected, 50.4% of healthcare workers experienced depression [8]. Globally, a systematic review and meta-analysis by Li et al. Reported an aggregate pooled prevalence of moderate depressive symptoms of 21.7% among healthcare professionals, noting that nurses and physicians accounted for 45% and 27% of the total evaluated population, respectively [9]. In Italy, symptoms were reported in 24.7% of cases [10]. In Turkey, a study evaluating healthcare workers reported that 77.6% exhibited depressive symptoms, with front-line deployment and female gender being two factors that significantly increased the psychological risk [11]. The same trend was observed significantly in the United States, where 48% of frontline healthcare workers experienced symptoms of depression [12].

The pandemic has affected family and work environments in unprecedented ways, contributing to negative changes in mental health that can have adverse effects on the quality of care and performance of healthcare professionals. Feelings of frustration, fear, and exhaustion stemming from the management and care of COVID-19 have left healthcare workers vulnerable, leading to an increase in depression [13]. Ciudad Juarez is located on Mexico’s northern border, directly adjacent to El Paso, Texas, and forms part of a highly interconnected binational metropolitan area. Research conducted during the COVID-19 pandemic examined cross-border mobility for healthcare and the use of both public and private health services in Ciudad Juarez, underscoring the distinctive health-service dynamics of this border setting [14]. This context provides a relevant framework for examining the mental health of frontline healthcare workers and for interpreting local findings alongside evidence for national and international healthcare systems with particular attention to the context of the Mexico–United States border region. The aim of this study is to describe depressive-symptom severity and explore its associations with sociodemographic and occupational characteristics among healthcare workers in a public hospital in Ciudad Juárez, Mexico.

## 2. Materials and Methods

A cross-sectional observational study was conducted at the public hospital of the Mexican Social Security Institute in Ciudad Juarez Mexico. This study aimed to estimate the prevalence of depression and examined associations with sociodemographic and occupational variables. In addition, statistical associations between factors and depression were explored; no causal or temporal relationships were assumed because of the cross-sectional design.

### Population and Sample

The target population consisted of frontline healthcare workers employed at the hospital during 2024 who met the inclusion criteria of having directly attended to patients with COVID-19. To ensure proportional representation across all shifts and job categories, a probability stratified random sampling method was utilized. The strata used for sampling were defined by professional roles (e.g., physicians, nurses, and allied health staff).

The sample size was determined based on an expected prevalence of depression of 20%, a 95% confidence level, and an expected precision (margin of error) of 5%. The denominator for the sampling frame consisted of 312 randomly selected and eligible healthcare workers who were invited to participate. With a 95% response rate, the final sample consisted of 296 healthcare workers.

Written informed consent was obtained, and participation was voluntary. Confidentiality was guaranteed; the study complied with the provisions of the Declaration of Helsinki and the General Health Law in Mexico. The study was approved by Local Health Research Committee number 801, under registration 2023-801-001 [15,16].

A questionnaire was developed that included sociodemographic and occupational variables for healthcare workers, such as age, sex, marital status, health conditions, job title, academic level, and access to personal protective equipment in the area. The Beck Depression Inventory II (BDI II) in the Mexican version was also used; it consists of 21 self-administered items designed to assess depressive symptoms and has been validated with a Cronbach’s alpha coefficient of 0.92. Scores range from 0 to 63, based on the severity and intensity of depressive symptoms: 0–13 = minimal depression, 14–19 = mild depression, 20–28 = moderate depression, and 29–63 = severe depression. The BDI II is a self-reported screening measure and does not, by itself, provide a clinical diagnosis of depressive disorder [17,18].

Data entry and descriptive univariate analysis were performed. Quantitative variables were expressed as means, and qualitative variables were expressed as frequencies and percentages. Bivariate analysis was performed using the chi-square test (*X*^2^), with statistical significance set at *p*-value < 0.05; odds ratios (ORs) and 95% confidence intervals (CIs) were also calculated. A database was created using the Statistical Package for the Social Sciences (SPSS, version 26). A multivariate binary logistic regression analysis using the enter method was performed to explore the relationship between the predictors and the severity of depressive symptoms. The proportion of explained variance and the overall strength of the model’s association were estimated using the Nagelkerke R-square coefficient. Statistical significance was defined as a *p*-value < 0.05 with an odds ratio (OR) > 1 and a 95% confidence interval (CI).

## 3. Results

There were 296 participants. The data showed a mean age of 35 years, 154 females, 38.2% married participants, and 66.2% with one or more children. Regarding work shifts, 42.6% worked the morning shift. Registered nurses and physicians comprised 34.5% and 32.4% of the sample, respectively, and 40.2% had a technical degree. Other variables included 10.8% of participants with health conditions and 63.5% who voluntarily isolated themselves to protect their families (Table 1).

The prevalence of depressive-symptom severity was 38.5%. General depression was associated with having a medical condition (OR = 4.11, *p* = 0.00001), anxiety (OR = 19.33, *p* = 0.00001), being female (OR = 1.96, *p* = 0.006), being in the 21–30 age group (OR = 1.66, *p* = 0.04), and voluntary self-isolated to protect one’s family (OR = 1.83, *p* = 0.01). Severe depression was associated with having hypothyroidism (OR = 37.06, *p* = 0.009) and being a specialist physician (OR = 2.92, *p* = 0.04). Moderate depression was associated with psoriasis (OR = 10.13, *p* = 0.01), rarely having access to the cafeteria during the workday (OR = 3.26, *p* = 0.004), occasionally having personal protective equipment (OR = 3.17, *p* = 0.005), not having personal protective equipment (OR = 2.78, *p* = 0.04), being on a cumulative shift schedule (OR = 3.29, *p* = 0.02), and being a general physician (OR = 2.57, *p* = 0.05). Mild depression was associated with being a nurse (OR = 1.98, *p* = 0.01), being female (OR = 2.00, *p* = 0.01), and voluntarily self-isolating to protect one’s family (OR = 2.08, *p* = 0.02) (Table 2).

In the logistic regression model, the most significant variables were anxiety (β = 3.02, *p* = 0.02), lack of personal protective equipment (β = 1.16, *p* = 0.03), and being a specialist physician (β = 1.04, *p* = 0.01). For severe depression, having hypothyroidism (β = 2.84, *p* = 0.05) was associated with a higher risk. In moderate depression, occasional use of personal protective equipment was significant (β = 1.46, *p* = 0.01) (Table 3).

## 4. Discussion

A high prevalence of depressive-symptom severity (38.5%) was identified among healthcare workers in Ciudad Juarez, Mexico. Other studies have identified a prevalence of depressive symptoms of 53.3% among physicians and 46.2% among nurses at a hospital in Peru [19]. Among frontline healthcare workers, a prevalence of 40.9% was identified in Paraguay [20]. In Argentina, depressive symptoms were found in 38.2% of physicians and 28% of nurses. In Bolivia, the prevalence was 24.2% among physicians and 27.3% among nurses. In Chile, the prevalence was 25.5% among physicians and 27.3% among nurses. In Colombia, the prevalence of depressive symptoms was 27.2% among physicians and 20.5% among nurses. In Ecuador, the prevalence was 27.1% among physicians and 26.9% among nurses [21]. Healthcare workers in the United States reported prevalence rates ranging from 19% to 43% across hospitals [22]. Healthcare workers on the front lines are experiencing mental health issues.

It has been reported that healthcare workers with comorbidities such as type 2 diabetes, high blood pressure, and thyroid disorders, among other conditions, are at increased risk of depression. This is consistent with our findings, which indicate that healthcare workers with a pre-existing condition were 4.11 times more likely to exhibit depressive symptoms in this sample [23]. Studies in Mexico have identified health risks among healthcare workers, such as being overweight or obese, smoking, and having chronic diseases, as risk factors for depression. Considering that healthcare workers with these conditions and associated risks are vulnerable, these factors may be associated with poorer mental health, particularly when they are exposed to patients with COVID-19 [24,25].

On Mexico’s northern border, information remains limited regarding working conditions, such as the protection and safety of frontline healthcare workers treating patients with COVID-19.

In the present Ciudad Juarez sample, voluntary self-isolation to protect family members was a constant source of concern, showing significant associations with depressive symptoms. Furthermore, in strict alignment with our statistical outcomes, a shortage or lack of personal protective equipment (PPE) was significantly associated with moderate depressive symptoms rather than severe presentations. In similar studies, voluntary isolation and the lack of information and essential equipment—such as masks, gloves, and protective suits—as well as the degree of exposure during working hours, the days and hours spent caring for patients, anxiety about the possibility of infecting family members, lack of personal safety, and long hours spent wearing protective clothing, among other factors, have been associated with depressive symptoms [24,26,27,28].

Work-related conditions, daily practices, and occupational categories among healthcare workers vary in their impact on mental health. Different levels of depressive symptoms have been associated with particularly high rates among physicians compared with nurses; however, some studies have identified a higher risk of severe symptoms among nurses. With the emergence of SARS-CoV-2, nursing staff experienced higher rates of depressive symptoms (24.3%); one study analyzed the impact on mental health during the COVID-19 pandemic and revealed that 45.3% of nursing staff suffered from these symptoms. In Spain, nursing staff were the most affected, at 53% [29,30,31]. This highlights the importance of and need for mental health support for all frontline workers in the fight against the pandemic [32].

It is important to emphasize that healthcare workers are not immune to depression; prevalence rates of depressive symptoms ranging from 19% to 68% have been identified. In China, the prevalence was 45.6%, and in Pakistan, 43%. This may be due to a lack of knowledge about the new disease, its rapid spread, and the fear of infection—risk factors that affect the mental health of healthcare professionals [33,34,35]. Regarding the higher prevalence of depressive symptoms among women, this vulnerability might be partially influenced by biological and hormonal factors regulating stress response mechanisms, as hypothesized in previous neuropsychiatry models [36]. However, this physiological aspect should be evaluated alongside the heavy work-related family burdens.

In the workplace, healthcare workers experience high levels of emotional exhaustion due to the increased demands of caring for patients with COVID-19. Female doctors and nurses working in healthcare in Mexico and Canada have higher levels of depressive symptoms than men; this may be due to a lack of physical safety, occupational exposure, and direct contact with patients, combined with the demands of raising children and serving as caregivers and, at times, experiencing physical and verbal abuse from patients’ family members. Women in Ecuador experienced higher rates of depressive symptoms during the pandemic. In some provinces of China, up to 87.1% of women experienced these symptoms due to working with COVID-19 patients [23,37,38,39]. In various articles, women outnumbered men by a ratio of 2 to 1, and it has been noted that the mental health impacts caused by this pandemic could exceed the number of people affected. Women experience higher levels of stress and distress and depressive symptoms due to the fear of personal infection or infection of a family member as a result of their work [39,40,41].

### Study Limitations

This study has several limitations. Because of its cross-sectional observational design, it was not possible to establish cause-and-effect relationships or the temporal direction of the observed associations; therefore, no causal inferences can be drawn regarding the development of depression. Furthermore, some exploratory estimates were based on participants’ self-reports, as the survey was self-administered and the subgroups were very small. Consequently, they had low statistical precision; these findings should be interpreted cautiously and confirmed in larger samples. The participating healthcare workers were not followed during the pandemic, and data collection took place several years after the end of the pandemic. Although overall the sample was adequate, future studies should include larger and more balanced cohorts, together with a comparison group of healthcare workers who did not work on the front lines during COVID-19, to estimate associations with depression more precisely.

Finally, the limited literature on depression among frontline healthcare workers caring for patients with COVID-19 on Mexico’s northern border restricts comparisons with similar studies.

## 5. Conclusions

The prevalence of depression symptoms among frontline healthcare workers was 38.5%, significantly higher among women than men. Rather than reflecting acute psychological distress during the COVID-19 crisis, these findings highlight a lingering post-pandemic psychological burden.

Chronic health conditions, alongside retrospective recollections of limited availability of protective equipment and supplies during occupational exposure, and past concerns about infecting family members, were significantly associated with current depression. However, because exposures were reported several years after the pandemic, they are subject to recall bias and must be interpreted strictly as retrospective factors.

These findings describe associations observed within this specific sample and should not be interpreted as evidence of causality. Further longitudinal and comparative studies, including healthcare workers without frontline COVID-19 exposure, are needed to clarify temporal relationships and strengthen the evidence for this border population.

## Figures and Tables

**Table 1:** Sociodemographic and occupational characteristics of healthcare workers.

Variables		
	mean	
Age	35	
**Age group**	frequency	percentage
18–20	2	0.7
21–30	101	34.1
31–40	122	41.2
41–50	59	19.9
>51	12	4.1
**Sex**		
Female	154	52.0
Male	142	48.0
**Marital Status**		
Married	113	38.2
Single	110	37.2
Divorced	34	11.5
Cohabiting	35	11.8
Widowed	4	1.3
Number of children		
No children	100	33.8
With children	196	66.2
**Do you have any health conditions?**		
Yes	32	10.8
No	264	89.2
**Diagnosed conditions**		
Anxiety	12	4.1
Hypothyroidism	3	1.0
Psoriasis	2	0.7
Others	15	5.0
Without illness	264	89.2
**Work Shift**		
Morning	126	42.6
Afternoon	99	33.4
Night	47	15.9
Compressed work schedule	24	8.1
**Job Title**		
Nursing Assistant	72	24.3
Registered Nurse	102	34.5
Clinical Nurse Specialist	19	6.4
Nurse Manager	7	2.4
General Physician	34	11.5
Specialist Physician	62	20.9
**Academic level**		
Technical degree	119	40.2
Bachelor’s degree	98	33.1
Specialist physician	73	24.7
Master’s degree	6	2.0
**Voluntary Self-Isolation to Protect Your Family**		
Yes	188	63.5
No	108	36.5
**Personal protective equipment available in the COVID-19 area**		
Did not have access	27	9.1
Occasionally had access	56	18.9
Had access	213	72.0
**Access to the cafeteria during the workday**		
Almost never	61	20.6
Occasionally	49	16.6
Had access	186	62.8

**Table 2:** Prevalence of depression symptoms across different levels.

	Number	Percentage	X^2^	*p* < 0.05	OR	Lower CI	CI Upper
**General depression**							
Anxiety	11	3.70	14.92	0.00001	19.33	2.46	151.87
Has a health condition	22	7.40	13.85	0.00001	4.11	1.86	9.05
Gender: Female	71	24.00	7.81	0.006	1.96	1.22	3.17
Self-isolation to protect her family	82	27.70	5.66	0.01	1.83	1.11	3.04
Age group: 21–30 years old	47	15.90	4.16	0.04	1.66	1.01	2.71
Did not have personal protective equipment	15	5.10	3.64	0.06	2.14	0.96	4.77
**Severe depression**							
Do you have any health conditions?	8	2.70	24.57	0.00001	9.44	3.33	26.72
Hypothyroidism	2	0.70	20.78	0.009	37.06	3.17	432.14
Anxiety	4	1.40	17.58	0.003	10.42	2.77	39.13
Specialist Physician	8	2.70	4.86	0.04	2.92	1.085	7.892
**Moderate depression**							
Psoriasis	2	0.70	17.21	0.01	10.13	7.17	14.32
Anxiety	5	1.70	12.98	0.004	7.08	2.10	23.92
Rarely had access to the cafeteria during the workday	13	4.40	9.62	0.004	3.26	1.49	7.11
Occasionally had personal protective equipment	12	4.10	8.84	0.005	3.17	1.43	7.00
Work shift: Cumulative workday	6	2.00	5.87	0.02	3.29	1.19	9.05
Has a health condition	7	2.40	4.97	0.03	2.80	1.09	7.14
Did not have personal protective equipment	6	2.00	4.37	0.04	2.78	1.03	7.55
General Physician	7	2.40	4.19	0.05	2.57	1.01	6.52
**Minimal depression**							
Age group: 21–30 years old	32	10.80	7.79	0.008	2.19	1.25	3.84
Occupation: Registered nurse	31	10.50	5.88	0.01	1.98	1.13	3.46
Gender: Female	43	14.50	5.86	0.01	2.00	1.13	3.53
Voluntary self-isolation to protect her family	50	16.90	5.49	0.02	2.08	1.11	3.87
Shift: Afternoon	29	9.80	4.20	0.05	1.79	1.02	3.14
Personal protective equipment provided	27	9.10	4.12	0.04	1.79	1.01	3.17
Education level: Bachelor’s degree	27	9.10	2.33	0.13	1.55	0.88	2.72

*p* = Significance. OR = Odds Ratio. CI = Confidence Interval.

**Table 3:** Multivariate binary logistic regression analysis of factors associated with depression symptoms.

	β	Sig.	Exp(β)	95% Confidence Interval for Exp(β)	
				Lower Limit	Upper Limit
**General depression**					
Anxiety	3.02	0.02	20.61	1.68	252.28
Did not have personal protective equipment	1.16	0.03	3.20	1.11	9.22
Specialist Physician	1.04	0.01	2.85	1.28	6.36
Occasionally had personal protective equipment	0.96	0.02	2.62	1.19	5.74
Registered nurse	0.82	0.01	2.27	1.19	4.30
Gender: Female	0.78	0.0001	2.18	1.30	3.64
Shift: Afternoon	0.70	0.02	2.02	1.13	3.62
Age group: 21–30 years old	0.56	0.04	1.76	1.03	2.99
General physician	0.75	0.14	2.13	0.78	5.84
Voluntary self-isolation to protect their family	0.45	0.10	1.57	0.91	2.69
Do you have any health conditions?	0.90	0.07	2.46	0.94	6.46
Hypothyroidism	1.36	0.38	3.91	0.19	79.55
**Severe depression**					
Hypothyroidism	2.84	0.05	17.14	1.96	305.53
Specialist Physician	0.93	0.09	2.55	0.86	7.54
Anxiety	1.30	0.19	3.69	0.53	25.58
Do you have any health conditions?	1.21	0.15	3.38	0.65	17.41
**Moderate depression**					
Occasionally had personal protective equipment	1.46	0.01	4.32	1.53	12.20
Did not have personal protective equipment	1.00	0.13	2.73	0.73	10.18
Work shift: Cumulative workday	0.89	0.15	2.43	0.72	8.22
Occasionally had access to the cafeteria during the workday	0.52	0.39	1.69	0.50	5.64
General physician	−0.14	0.81	0.82	0.25	2.92
**Minimal depression**					
Gender: Female	0.64	0.03	1.90	1.05	3.45
Age group: 21–30 years old	0.55	0.09	1.74	0.92	3.27
Self-isolation to protect her family	0.53	0.11	1.70	0.89	3.25
Work shift: Afternoon	0.50	0.09	1.65	0.92	2.98
Registered nurse	0.48	0.11	1.62	0.89	2.92
Had personal protective equipment	0.33	0.28	1.39	0.76	2.57
Education level: Bachelor’s degree	0.16	0.61	1.17	0.62	2.22

β = Beta. Sig. = Significance. Exp (β) = Beta exponential.

## Data Availability

The data presented in this study are available upon request from the corresponding author (Data are not publicly available due to privacy or ethical restrictions).
